# Pupil Unleashed: Unraveling the Enigma of an Unusual Traumatic Head Injury: A Case Report

**DOI:** 10.5811/cpcem.20308

**Published:** 2024-08-01

**Authors:** Akash Daswaney, Shuchi Abhishek, Sanjan Asanaru Kunju, Priya Pattath Sankaran, Ahlam Abdul Rahman

**Affiliations:** * Department of Emergency Medicine, Kasturba Medical College, Manipal, Manipal Academy of Higher Education, Manipal, Karnataka, India; † Kasturba Medical College, Manipal, Manipal Academy of Higher Education, Manipal, Karnataka, India; ‡ Department of Emergency Medicine, Kasturba Medical College, Mangalore, Manipal Academy of Higher Education, Manipal, Karnataka, India; § Department of Radiodiagnosis and Imaging, Kasturba Medical College, Manipal, Manipal Academy of Higher Education, Manipal, Karnataka, India; ∥ Department of Anaesthesiology, Kasturba Medical College, Mangalore, Manipal Academy of Higher Education, Manipal, Karnataka, India

**Keywords:** *oculomotor nerve palsy*, *minor head trauma*, *emergency department*, *case report*

## Abstract

**Introduction:**

Isolated oculomotor nerve palsy after mild traumatic brain injury is unusual and prognostically significant due to unclear mechanisms and recovery challenges. We present a case of isolated oculomotor nerve palsy following minor head trauma, shedding light on this unusual occurrence.

**Case Report:**

A 24-year-old male experienced severe vision loss and right-sided oculomotor nerve palsy after a motor vehicle collision. Initial imaging showed a hemorrhagic focus in the left posterior fossa and a contusion in the corpus callosum, yet no direct cause for the nerve palsy was found. Partial recovery was observed after 12 months.

**Conclusion:**

This case underscores the importance of maintaining a heightened suspicion for occult intracranial findings, especially when the initial non-contrast computed tomography was inconclusive. Timely clinical assessment and appropriate radiological investigations by emergency physicians are crucial for improving the prognosis.

CPC-EM Capsule What do we already know about this clinical entity?

*Isolated oculomotor nerve palsy following mild traumatic brain injury (TBI) is rare, and indirect injuries predominate.*

What makes this presentation of disease reportable?

*This case underscores isolated oculomotor nerve palsy post-mild TBI, prompting re-evaluation due to absence of focal deficits and correlating radiological findings.*

What is the major learning point?

*Precise imaging enhances oculomotor nerve palsy management. Thorough assessment and tailored treatment are vital in mild TBI cases.*

How might this improve emergency medicine practice?

*Thorough clinical assessment and imaging, precise strategies, and advocating standardized evaluation across injury severities elevate emergency medicine standards.*

## INTRODUCTION

Isolated oculomotor nerve palsy following mild head injury is a rare occurrence. The mechanism required to damage the nerve is usually severe and is often associated with basilar skull fracture, orbital injury, subarachnoid hemorrhage, or neurological deficits such as loss of consciousness.^1^ Injury to the nerve could be direct due to shearing force between the brainstem and supratentorial structures causing rootlet avulsion and distal fascicular damage or indirect, caused by compression, displacement, or deformity of the nerve due to a space-occupying lesion like an expanding hematoma.^2^ It is often described to occur following an expanding intracranial mass lesion, causing uncal herniation.^3^ The recovery rate, in terms of sympathetic and parasympathetic oculomotor function, following complete oculomotor nerve palsy is prolonged, often resulting in incomplete resolution. In this case, we describe a patient who developed isolated right oculomotor nerve palsy following minor closed head trauma (Glasgow Coma Scale [GCS]15). A peculiar feature was the lack of association between the magnetic resonance imaging (MRI) findings and clinical manifestations that gave us insight into the injury mechanism to the nerve.

## CASE REPORT

A previously healthy 24-year-old helmeted male, motorbike rider presented to the emergency department (ED) of a tertiary care center after a road traffic accident. Unable to control the speed of his motorcycle, he skidded and fell while taking a sharp turn. On admission, he had severe pain over his right shoulder and hip, diminution of vision on the right side, inability to open his right eye, right-sided diplopia, and inability to recollect any events surrounding the incident. He denied loss of consciousness, nose bleeding, vomiting, or seizures.

**Image 1.**
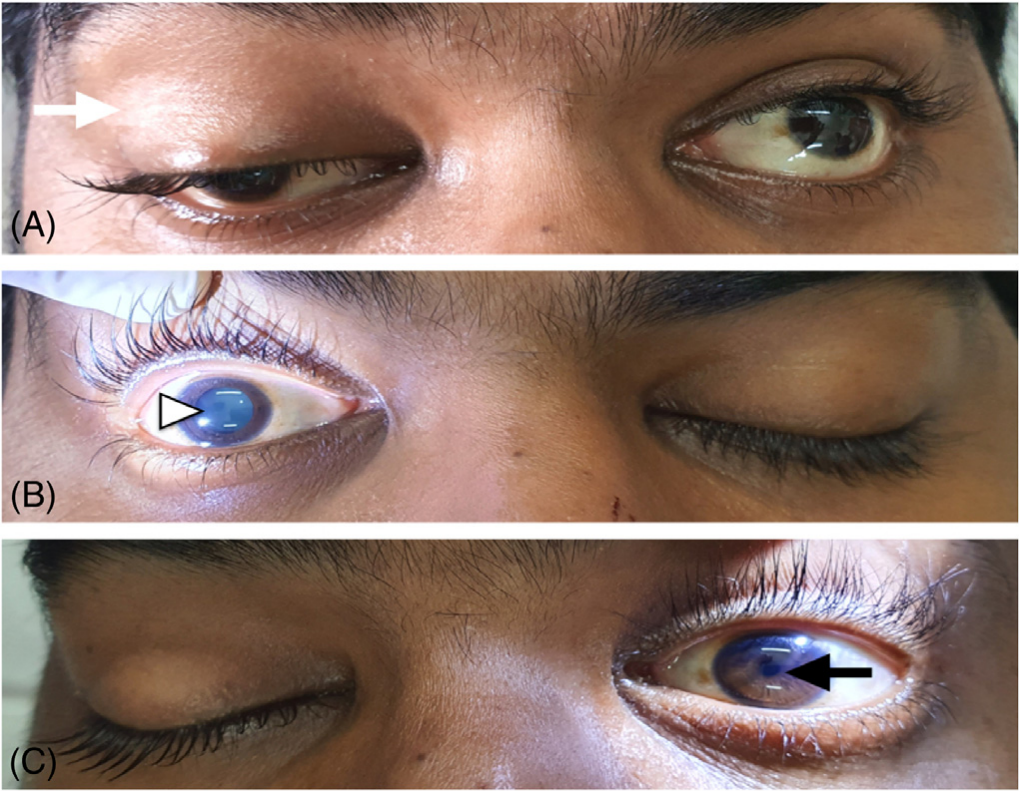
Photograph of the patient that showed features of oculomotor nerve palsy on right side: a) ptosis (white arrow), indicative of involvement of the levator palpebrae superioris (sympathetic involvement); b) absent pupillary light reflex—‘“fully blown out dilated pupil’”(white arrowhead) indicative of involvement of the sphincter pupillae (parasympathetic involvement); c) intact left-sided pupillary light reflex (black arrow).

At presentation, he was conscious with a GCS score of 15. The vitals recorded were heart rate 90 beats per minute, blood pressure 140/90 millimeters of mercury (mm Hg), respiratory rate 16 breaths per minute, 100% oxygen saturation on room air, and afebrile. Initial neurological examination showed that he was cooperative, alert, and well oriented to person, place, and time. There was ptosis, lateral deviation of the eyeball, and a fixed, dilated, and non-reactive pupil measuring 7 millimeters (mm) on examining the right eye. He was unable to adduct, elevate, or depress the right eyeball (Image 1). Orbital margins were intact. The conjunctiva appeared normal, the cornea was clear, the anterior chamber looked normal, and the lens was transparent. The left eye appeared normal with no limitation in extraocular movements, and the pupil was 2 mm and reactive. All other ophthalmic examinations, including measurement of intraocular pressure, revealed normal findings. Examination of other cranial nerves and systemic examination was unremarkable.

**Image 2.**
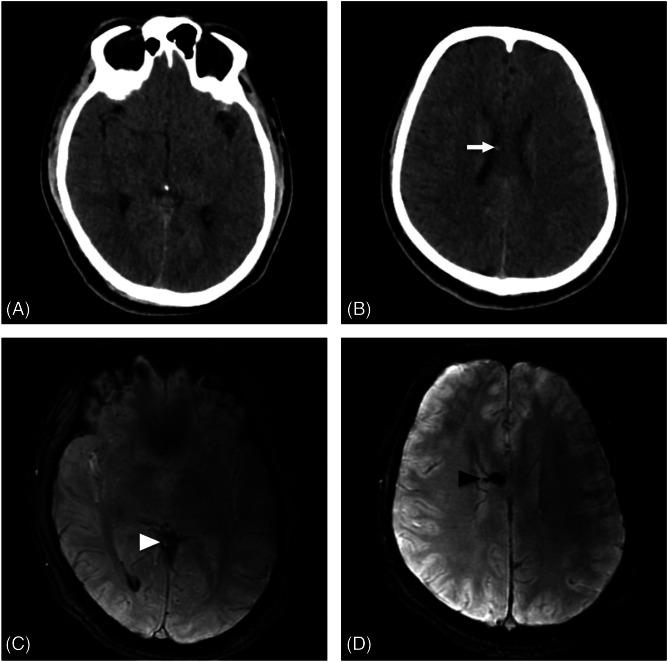
Non-contrast computed tomography brain of a 24-year-old male (axial view): a) without any evidence of Intraparenchymal injury; b) showing tiny hemorrhagic foci (white arrow). Magnetic resonance imaging of the brain (axial 3-D susceptibility-weighted angiography) at the corresponding section showed; c) blooming foci in the posterior horn of the right lateral ventricle (white arrowhead) suggestive of minimal intraventricular hemorrhage; d) blooming foci in the body of corpus callosum on the right side (black arrowhead), measuring 6.0 × 8.0 mm—likely minor hemorrhagic contusion.

In this case, owing to the intricate nature of the patient’s presentation characterized by isolated oculomotor nerve palsy after mild head trauma, a multidisciplinary team comprising experts from emergency medicine, neurology, ophthalmology, and radiology collaborated to deliver meticulous and comprehensive care. Laboratory examinations were normal except for an elevated white blood cell count. Radiographs of the shoulder and hip on the right side revealed a clavicle fracture and an intertrochanteric fracture of the femur, respectively. Non-contrast computed tomography (CT) of the brain and orbits revealed a well-defined hemorrhagic focus measuring ∼ 0.9 × 2.2 centimeters in the left posterior fossa that did not correlate to the observed clinical findings (Image 2). The patient was transferred to the intensive care unit for further monitoring, and a multitude of additional diagnostic scans were performed. Magnetic resonance imaging of the brain revealed a small hemorrhagic contusion over the body of the corpus callosum on the right side and minimal intraventricular hemorrhage in the posterior horn of the right lateral ventricle (Image 2).

**Image 3.**
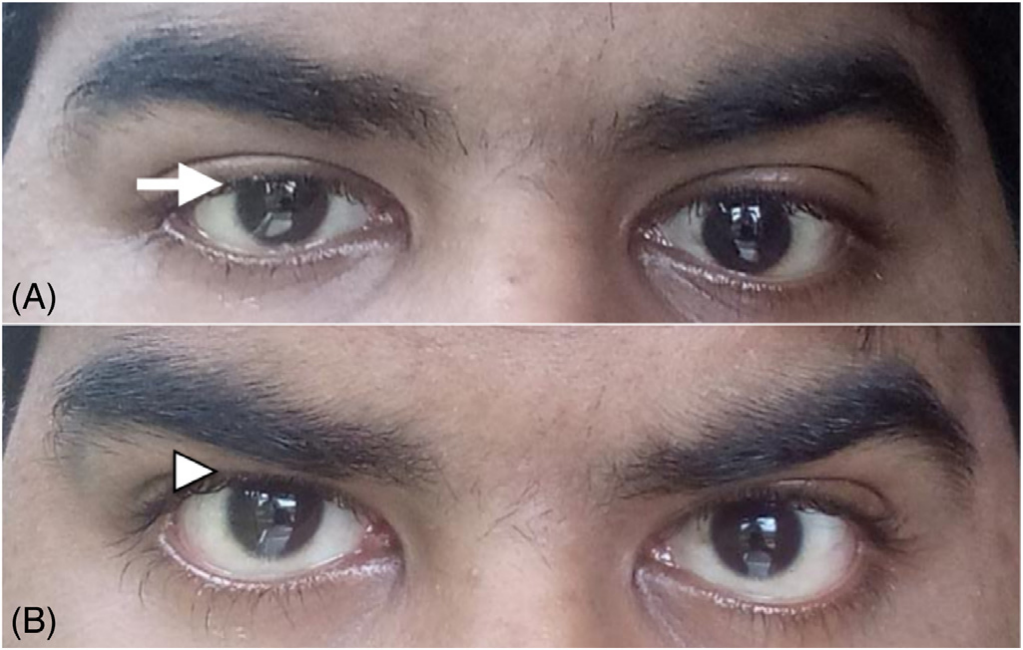
Photograph of the same patient taken 10 months after the injury: a) persistence of mild ptosis (white arrow) in the right upper lid at rest; b) improvement of ptosis (white arrowhead) on raising the eyebrows.

An ophthalmologist recommended to instill antibiotic (to prevent a superimposed bacterial infection) and lubricant eye drops in the right eye and to do cold compresses over the right eye three times a day for one week. A repeat evaluation two days later showed the pupil size to be 5 mm, fixed, and dilated. This was an improvement compared to the 7 mm noted in the ED. In addition, the fundus examination of the right eye was normal. He was advised to continue the same treatment and was discharged after nine days. Over the next 12 months, he experienced partial improvement in some aspects of his right oculomotor function (Image 3).

## DISCUSSION

A patient who presents with blurring of vision, headache, diplopia, ptosis, evidence of trauma, and focal neurological deficits points an emergency physician to a potentially alarming situation. Due to this diverse presentation and the vast differential, an oculomotor nerve palsy encounter should begin with a thorough history, physical examination, and radiological investigation.

Traumatic insults (eg, traumatic brain injury [TBI], subdural hematoma, basilar skull fracture, brain herniation syndromes), autoimmune exacerbations (eg, multiple sclerosis, myasthenia gravis), vascular anomalies (eg, cerebrovascular accidents, posterior communicating artery aneurysm, basilar artery aneurysm, subarachnoid hemorrhage), neoplasms (eg, pituitary apoplexy, skull-based tumors), and infections (eg, orbital cellulitis predisposing a cavernous sinus thrombosis) represent the emergent spectrum of third cranial nerve palsies.^4^

Previous studies have employed “the rule of the pupil” to determine how radiological investigations are expedited in such cases.^5^ The subarachnoid location of the pupillary constrictor fibers makes them easily compressible by posterior communicating or basilar artery aneurysms. Therefore, the lack of pupil involvement would often point to a less emergent condition such as ischemia secondary to diabetes mellitus. Similarly, frequent physical examinations and liberal consultations lead to clinical clues (unilateral vs bilateral involvement, immediate vs delayed presentation, complete vs partial extraocular dysfunction, signs of basilar skull fracture, focal neurological deficits, Cushing triad) that help rule out emergent conditions.^6^

In our case, the patient presented with an isolated right-sided oculomotor nerve palsy following a mild TBI. The absence of accompanying red flags, signs, or symptoms and the lack of explanatory radiological findings make this case unique. Contrastingly, in both the Solomons et al and Memon et al studies, no isolated third nerve palsy cases were caused by mild head injury.^3^ ^,^ ^7^ Only 0.04% of traumatic oculomotor nerve palsy had been reported by a study done in 19,800 mild head injury cases. In our case, the impact was a direct right-sided frontal blow with the radiographic finding that does not correlate with the clinical presentation of the TBI.^8^

However, the exact mechanism of isolated oculomotor nerve palsy caused by mild TBI is not precise. The injury to the oculomotor nerve can occur anywhere along its course from the midbrain to orbit.^4^ Three critical parts where it can be damaged are at the site where the nerve emerges from cerebral peduncles, upon entering the cavernous sinus, and within the cavernous sinus. While indirect injuries (resulting from a disturbance in blood supply or detrimental biomechanical effects following a head injury) have been reported to have a higher incidence than direct injuries (mechanical damage), information is scarce on inconsequential third nerve palsy management in such settings. Furthermore, such patients would have delayed presentation, which was not the case in this scenario.^2^

According to Kaido et al, a rostrocaudal line of force is generated following a frontal blow. The direction is parallel to the third nerve and can cause traction at fixation points, causing stretching and intraneural hemorrhage.^9^ The “open V formation” of both oculomotor nerves likely prevents bilateral direct third nerve injury, and that is why it is rarely observed clinically.^7^ A shock wave generated at the time of impact can also cause damage to the third nerve.^10^ Diffusion tensor imaging and fiber tractography are recent imaging techniques that could assess white fiber damage and may help us in diagnosing, predicting, and prognosticating oculomotor nerve palsy.^11^

It is uncommon to find a traumatic cause of isolated oculomotor nerve palsy without correlating radiological findings. Multipositional, high-resolution MRI with T1- and T2-weighted images in most patients with suspected third nerve palsy may show abnormal enhancement of the oculomotor nerve because of ischemia, inflammation, or demyelinating conditions.^1^ A few studies reported radiological evidence of injury to the nerve as it passes over the tough petroclinoid ligament.^9^ ^,^ ^12^ In contrast to the radiological features present in these studies, our findings on MRI included a minor hemorrhagic contusion over the body of the corpus callosum on the right side and minimal intraventricular hemorrhage in the posterior horn of the right lateral ventricle that neither revealed the cause nor location of the injury. The lack of relation between imaging abnormalities and observed clinical findings gave us an insight into the other probable mechanisms of third nerve injury in mild TBI.

Based on current evidence, the appropriate management of isolated oculomotor nerve palsy caused by mild TBI is still unclear. Our recommendation would be, to begin with, a non-contrast CT head to determine the need for immediate surgical intervention (evacuation via a craniotomy or burr hole or resection of a tumor) with or without steroids.^1^ If inconclusive, a three-dimensional MRI of brain and blood vessels (including T1- and T2-weighted images, magnetic resonance angiography, and diffusion-weighted images) should be considered. In addition to this, Felix et al suggested an isotropic, high-resolution, T2-weighted constructive interference steady state (CISS) sequence and contrast-enhanced, axial and coronal T1 sequences to detect direct and indirect mechanisms of injury.^13^

Similar to the treatment rationale in patients with delayed facial nerve palsy, some studies have suggested using steroids as it reduces endoneurial edema to prevent secondary neuronal damage.^1^ ^,^ ^14^ As opposed to other cases, there was no indication for surgical treatment in our patient. Observation and follow-up are the cornerstones of management in patients with no definite intracranial findings. A similar strategy was adopted in our case: instilling antibiotic and lubricant eye drops five times a day and cold compress every eight hours. The pupil size had reduced from 7 mm to 5 mm on the fourth day, indicating slight improvement. A few reports introduce reading-related neurorehabilitation like facial massage and eye-tracking exercises and injecting botulinum toxin into the lateral rectus muscle to prevent contracture and may reduce the morbidity.^1^ ^,^ ^2^

The prognosis of third nerve palsy is generally poor, with full recovery being uncommon, resulting in high morbidity.^15^ Ptosis has been reported to resolve earlier than impaired extraocular muscle movement, while the pupillary size and light reflex show the least degree of recovery following the course of events. Although the time interval to resolution varied, our review revealed that patients who had mild TBI with initial GCS scores of 13 or more experienced at least partial resolution. Our patient experienced at least partial improvement in some aspects within 12 months (Image 3).

## CONCLUSION

The presence of fixed and dilated pupils following TBI in the emergency department has been associated with high mortality. However, we highlight the occurrence of isolated third nerve palsy in mild TBI with no other focal neurological deficits through this case. Although the appropriate management of isolated oculomotor nerve palsy caused by mild TBI is still unclear based on current evidence, a review of management and outcomes helps establish goal-oriented treatment plans and rehabilitation. Moreover, we stress the importance of a comprehensive clinical assessment. Also, we recommend radiological imaging tests regardless of injury severity to assess the cause of damage to plan for treatment and improve prognostic outcomes.
